# Communication phrases to support trauma-informed care in oral health: a concise review

**DOI:** 10.1038/s41415-026-0044-6

**Published:** 2026-10-23

**Authors:** Kristy S. Choi, Woosung Sohn, Delyse Leadbeatter

**Affiliations:** School of Dentistry, Faculty of Medicine and Health, University of Sydney, Australia

## Abstract

**Introduction** Effective communication is essential in providing quality patient care and the learning of communication phrases can be helpful with unfamiliar or challenging clinical situations. Trauma-informed care (TIC) is a patient care framework to support patients with trauma and the practice of TIC in oral health (OH) is limited and can be challenging for OH practitioners. The aim of this review is to identify communication phrases to practise TIC in OH.

**Method** A literature search was conducted through six electronic databases for studies published between January 1992 to September 2025 with consistent search terms. Grey literature was searched through Google Scholar and citation searching of the identified papers.

**Results** The search identified 167 papers and nine grey literature articles; 27 papers on TIC in OH were identified and screened in full-text which resulted in eight papers with communication phrases when practising TIC. Identified phrases were categorised according to clinical situations: before the treatment, screening for trauma, during the treatment, when trauma signs and symptoms are recognised, when trauma history is disclosed, active listening and motivational interviewing.

**Conclusion** Communication phrases could be a practical communication tool in supporting OH practitioners when practising TIC but needs further research to validate its effectiveness.

## Key points

- Trauma-informed care is an emerging patient care framework to support patients with trauma, and clinical communication is a foundational component of trauma-informed care enabling oral health practitioners to foster safety, trust, control, collaboration and empowerment.
- This review identified communication phrases which can be used as a valuable communication tool to support the practice of trauma-informed care in oral health.
- Communication phrases can improve patients' experience in oral health and reduce oral health practitioners' burden of practising unfamiliar trauma-informed care, but its effectiveness warrants further research.

## Introduction

Effective communication in healthcare is crucial for ensuring positive health outcomes, enhancing patient satisfaction and practitioners' wellbeing.^1^^,^^2^ The importance of communication in healthcare is highlighted by its role in fostering understanding, compliance, and trust, which are essential for effective diagnosis, treatment, and patient safety.^3^^,^^4^^,^^5^ In oral health (OH), effective communication can play a pivotal role in managing patients' dental anxiety^6^^,^^7^ and building trusting relationship between OH practitioners and the patients, enabling successful delivery of person-centred care in OH.^8^

In healthcare, scripting refers to using predetermined, structured templates or communication phrases for certain interactions to deliver thoughtful and appropriate responses.^9^ While understanding communication principles and theories provide a valuable foundation for clinical communication, communication phrases can be a practical communication tool in clinical practice. Consistent communication phrases between patients and nurses led to a significant improvement in patient satisfaction with pain management over time^10^^,^^11^ and the use of communication phrases has been shown to significantly reduce elopement rates in emergency patients.^12^ Employing communication phrases in healthcare was shown to be helpful when handling difficult situations and serve as the foundation for starting or redirecting a conversation.^13^^,^^14^ When executed well, communication phrases can be used to demonstrate respect and empathy, improving patients' sense of empowerment and engagement for optimised treatment outcomes.^15^

Trauma-informed care (TIC) has gained increasing attention as a patient care framework to support patients with trauma and to prevent re-traumatisation.^16^ One can develop trauma from experiencing traumatic events such as violence and abuse.^16^ Trauma may arise from exposure to adverse experiences, including violence and abuse, which are not limited to physical forms but may also be emotional or psychological in nature. These experiences can have significant health consequences, including impacts on oral health (OH). Such effects are often less readily identifiable than the overt signs of physical injury observed in patients presenting to clinical care.

There is high prevalence of traumatic events in our society.^17^ Given the prevalence of orofacial injuries associated with violence^18^ and the multifaceted impact of trauma on OH,^19^^,^^20^ OH practitioners are highly likely to encounter patients with trauma. Patients with trauma can present with various challenges such as dental fear and anxiety, compromised OH and difficulty in forming trusting relationships with OH practitioners.^21^^,^^22^^,^^23^ TIC can be conceptualised as a ‘universal precaution'. As standard infection control is applied to all patients regardless of known status, TIC can be applied universally, providing a supportive environment to safeguard both patients and practitioners from distress and re-traumatisation.^24^ However, there remains a gap in how TIC concepts and principles are applied by OH practitioners in day-to-day encounters with patients.^25^^,^^26^

TIC emphasises principles of safety, trust, control, collaboration and empowerment.^16^^,^^27^ While theoretical underpinnings of TIC principles may have been introduced, TIC practice is limited in OH,^25^ many OH practitioners do not feel sufficiently skilled,^22^^,^^28^ and they are rarely taught how to communicate when practising TIC,^29^ highlighting the need for enhanced training practise, and a supportive framework to build confidence. Communication skill is a critical component of TIC^29^^,^^30^ and the absence of specific TIC communication tools may increase the risk of re-traumatisation and unnecessary distress for both patients and OH practitioners. Learning about patients' safety and trust is not the same as knowing what to say when a patient becomes anxious in the chair or discloses a trauma history. TIC communication phrases would be highly beneficial for patients and OH practitioners, offering a structured and accessible way to translate TIC theory into practice.

As per the knowledge of the authors of the present paper, there are no literature-informed recommendations about communication phrases when practising TIC in OH. Thus, extracting key communication phrases from literature that OH practitioners could use could build tangible TIC competence in OH practitioners. Hence, the aim of this study was to investigate and identify communication phrases from literature to support the practice of TIC in OH.

## Method

A comprehensive literature search was done through the electronic databases; Medline via Ovid, Embase via Ovid, CINAHL, Scopus, ProQuest and Web of Science for studies published in English between January 1992 to September 2025 to capture all TIC studies in OH since the foundation of The Substance Abuse and Mental Health Services Administration (SAMHSA), an agency within US Department of Health and Human Services that pioneered TIC since 1992.

Following search terms were used (trauma-informed OR ‘trauma sensitive' OR ‘trauma responsive' OR ‘trauma focused') AND (‘dental health*' OR ‘oral health*' OR ‘oral care*' OR ‘dental care*' OR dentistry OR dentist* or ‘dental profession*' OR ‘dental practice*'). The final search was completed on September 24 2025. Ethical approval was not required for this review of existing literature.

## Results

**Fig. 1:**
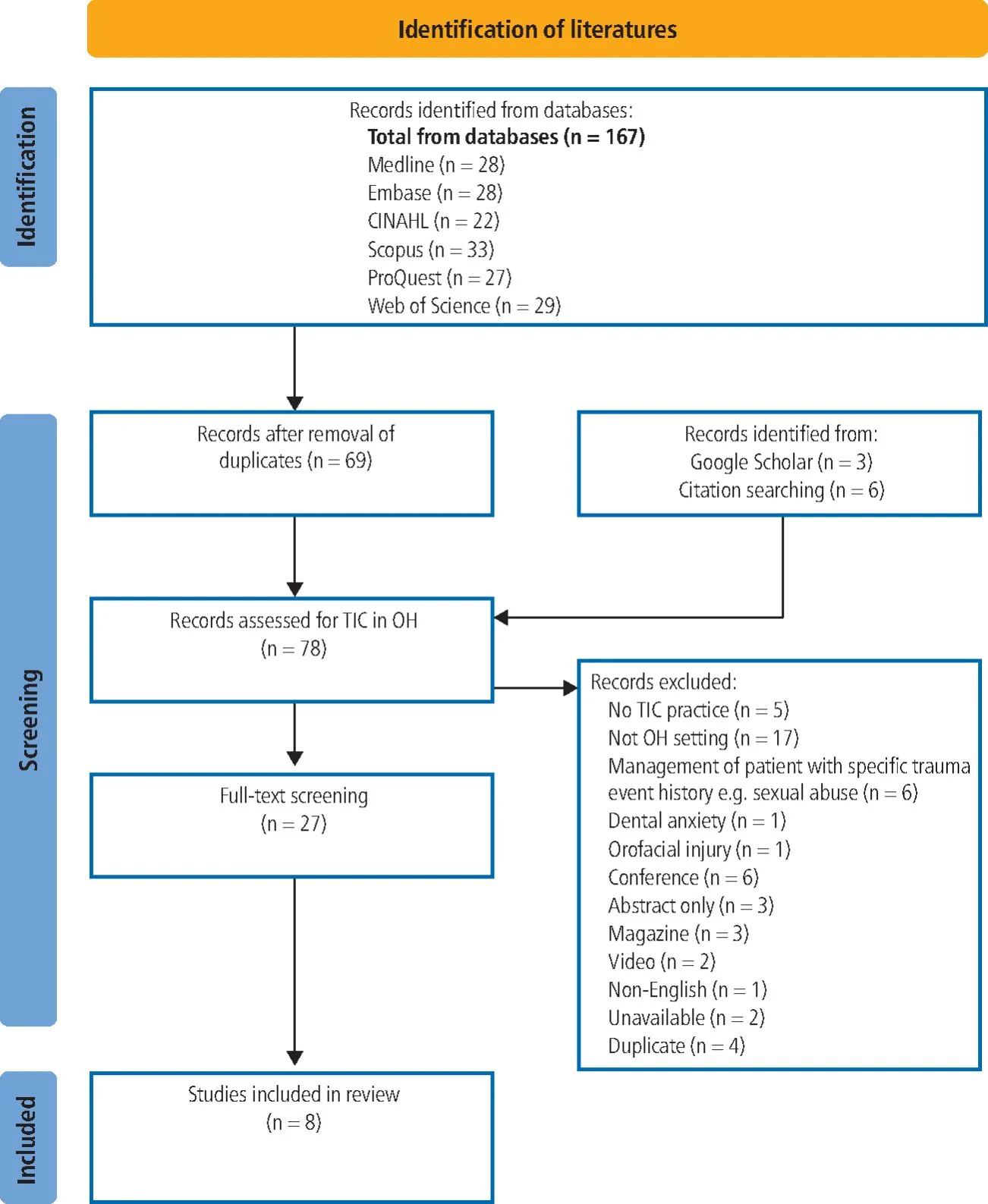
Search flowchart

This review identified 167 records through database searching. After removal of duplicates, 69 records remained, resulting in a total of 78 papers, including nine sources of grey literature. Out of 78 papers, 27 papers discussed TIC in OH and 27 papers were screened in full-text to identify eight papers with communication phrases for practising TIC in OH (Fig. 1). A descriptive summary of eight papers is provided in Table 1. Communication phrases were categorised according to clinical situations: before the treatment, screening for trauma, during the treatment, when trauma signs and symptoms are recognised, when trauma history is disclosed, active listening and motivational interviewing (Table 2). Identified communication phrases are summarised in Table 3.

**Table 1:** Descriptive summary of the papers

**No.**	**Author/online access**	**Title**	**Journal/Source**	**Article type**	**Country**	**Population**	**Summary/main findings**
1	Anstorp *et al.*^25^ -3-031-04248-5_2	The importance of trauma-sensitive care	*Oral Health Psychology*, Textbooks in contemporary dentistry	Textbook chapter	Switzerland	Dentists and hygienists	Links ACEs to lifelong OH impacts and suggests using ‘window of tolerance' model to provide trauma-informed and trauma-responsive care in dental setting.
2	Gunter *et al.*^27^ https://doi.org/10.1038/s41415-023-5758-0	Top tips for supporting patients with a history of psychological trauma	*British Dental Journal*	Commentary	UK	All members of dental work force	Presents how to recognise psychological trauma symptoms with ‘window of tolerance' model and management of dissociation. Includes five principles of TIC and self-care strategies for the dentists.
3	Hammett *et al.*^29^ https://www.wadvocates.org/wp-content/uploads/2020/01/Trauma-Informed-Care-and-Oral-Health.pdf	Trauma-informed care and oral health: recommendations for practitioners	Illinois ACEs Responsive Collaborative/Health and Medicine Policy Research Group	Association publication	USA	Oral health providers	Describes TIC in OH as essential practice for providing sensitive and effective treatment to patients who have experienced trauma. OH practitioners can enhance patient trust and engagement by integrating TIC principles into their practice.
4	Heaton and Cheung^24^ https://jdh.adha.org/content/98/4/50	Trauma-informed care in oral health care: the role of dental hygienists	*Journal of Dental Hygiene*	Commentary	USA	Dental hygienists	Highlights the critical role of dental hygienists in providing TIC, emphasising its impact on patient comfort, oral health outcomes, and trust.
5	Lapidos *et al.*^31^ https://doi.org/10.1080/19424396.2024.2353739	Behavioural health in dental settings: resources to support integration	*Journal of the California Dental Association*	Literature review	USA	Dental professionals	Advocates for integrating behavioural health and OH, emphasising universal trauma precautions and evidence-based communication to address the bidirectional link between mental health and oral health outcomes.
6	Magee^28^ https://mydigimag.rrd.com/article/A+Trauma-Informed+Approach+to+Dental+Care/3366037/583212/article.html	A trauma-Informed approach to dental care	*Journal of Massachusetts Dental Society*	Case study	USA	Dentists	TIC in dentistry exploring understanding trauma's impact on patients, employing patient-centred communication, and creating a safe environment to improve OH outcomes.
7	Raja *et al.*^19^ https://doi.org/10.14219/jada.2013.30	Treating patients with traumatic life experiences providing trauma-informed care	*Journal of the American Dental Association*	Commentary	USA	Dentists	TIC pyramid is proposed to help dentists to engage with traumatised patients in OH care, based on communication skills, understanding the effects of trauma, collaboration, self-awareness and screening.
8	Swarthout^26^ https://doi.org/10.14219/jada.2013.30	Trauma-informed approach to care in the dental setting	*Dental Academy of Continuing Education*	Educational document	USA	Dental health care providers	TIC introduction on communication skills, understanding trauma's health impact, interprofessional collaboration, and provider self-awareness to improve patient engagement and outcomes.
ACEs, adverse childhood experiences; OH, oral health; TIC, trauma-informed care.

**Table 2:** Papers categorised into clinical situations

		**During appointment booking**	**Before the treatment**	**During the treatment**	**Screening for trauma**	**Trauma signs and symptoms are recognised**	**Trauma history is disclosed**	**Active listening**	**Motivational interviewing questions**
1	Anstorp *et al.*^25^				*	*			
2	Gunter *et al.*^27^		*			*		*	
3	Hammett *et al.*^29^		*						
4	Heaton and Cheung^24^						*		
5	Lapidos *et al.*^31^					*	*		
6	Magee^28^		*		*				
7	Raja *et al.*^19^		*	*	*		*		
8	Swarthout^26^								*

**Table 3:** Trauma informed care communication phrases

**Clinical context**	**Phrases**
Before the treatment	‘What can I do to make you more comfortable during the procedure or examination today?'^22^ ‘ls there anything we can do to make you feel more comfortable?'^31^^,^^32^ ‘Before we proceed, is there anything else you think I should know?'^22^ ‘Is anything about today's appointment worrying you?'^32^ ‘Just to let you know, this is generally how this type of appointment runs. First, I will get a history, then we will take a look in your mouth, and then we will take x-rays. I'll come back to talk and answer questions.'^22^ ‘Here are the steps to the procedure we'll be performing. Is it alright if I place this instrument in your mouth?'^33^ ‘Has listening to music during a procedure helped you get through this type of procedure before?'^22^ ‘Is there anything you can visualize to help you relax?'^22^
Screening for trauma	‘Do you feel safe at home?'^22^^,^^32^ ‘Is anyone making you feel unsafe right now?'^22^ ‘Do you have a safe place to go when you leave this appointment?'^22^ ‘Is anyone hurting you?'^22^ ‘Sometimes when I see injuries like this, they are the result of someone's hurting someone else. Can you tell me if that applies here?'^22^ ‘Does your reaction today (… describe) have something to do with experiences from the past?'^29^
During the treatment	‘I am going to do a percussion test on your tooth. To do this, I will gently tap your teeth. Let me show you on my hand, or we can use yours. It will feel like this [tap on hand]. Let's try it on your tooth.'^22^
When trauma signs and symptoms are recognised	‘It is common to have a stress reaction or to experience anxiety in our setting. How can we make this experience better for you?'^35^ ‘I may not really understand what you're going through, but I am here to support and listen to you.'^35^
	**When dissociation identified** ‘You are here with me, the dentist, now. Please come back here.' ‘Is this a reaction you can recognise from other situations as well? Did you notice the feeling right before you disappeared? What can we do to help you not fade away, but to stay more present here with me?'^28^ ‘Susan is something happening right now?'^28^ ‘Are you able to hear me?'^28^ ‘You are (NAME), you are in the dental surgery with (PRACTITIONER NAME/anyone else who is present).'^28^ ‘You are not in danger.'^28^
When trauma history is disclosed	‘I want to thank you for sharing your experience and trusting me with your story. It sounds like that was a really difficult (frightening, stressful; mirror the patient's descriptive words here) situation, and I'm sorry to hear you went through that. It makes sense that, having gone through that, coming to the dental office can also be difficult (again, mirror the patient's words). We all want you to be able to feel more comfortable receiving care here. What are some things you think might be helpful to make you feel more comfortable receiving dental care going forward? Is there anything you would like us to be sure to do, or try to avoid doing if we can?'^28^ ‘I am sorry that happened to you. How are you doing now?'^22^^,^^35^ ‘Thank you for telling me. Is there anything I can do as your dentist to make you feel more comfortable?'^22^^,^^35^ ‘I appreciate your telling me that. No one deserves that to happen to them.'^22^^,^^35^ ‘Do you think any of these experiences affect you today? If so, would you like a referral to talk more about some of these issues?'^22^^,^^35^
Active listening	‘What I've heard you say is…'^31^ ‘So what I understand is…'^31^ ‘Is this right?'^31^ ‘Have I understood you correctly?'^31^
Motivational interviewing question	‘On a scale of 1–10, how important is it to you to improve the health of your gums?'^30^

### Before the treatment

A sense of safety and trust is a crucial component of TIC and OH practitioners can create this environment before the treatment by asking about a patients' level of comfort, obtaining explicit consent for any actions, explaining steps involved in the treatment, providing control to stop the procedure when needed, and explaining the expected outcome of the treatment. Four papers provided questions OH practitioners can ask before the treatment to practise TIC and screen a patient's level of comfort.^22^^,^^31^^,^^32^^,^^33^ According to Hammet *et al.*,^33^ continuous use of consent-based language can help create sense of safety and trust for patients. Explaining steps of upcoming actions can also build a sense of trust and support informed decision making.^22^^,^^33^ Raja *et al.*^22^ highlight the importance of asking questions to identify past coping mechanisms and support patients who may be anxious or fearful.

### Screening for trauma

Three papers provided communication phrases when asking general and indirect questions to screen for trauma.^22^^,^^29^^,^^32^ When patients present with acute orofacial injuries such as visible bruising, broken bones or lacerations, OH practitioners can ask more direct questions such as ‘is anyone hurting you?'^22^ or ‘sometimes when I see injuries like this, they are the result of someone's hurting someone else. Can you tell me if that applies here?'.^22^ When unexpected reactions arise during the treatment, OH practitioners can also ask questions such as ‘does your reaction today (… describe) have something to do with experiences from the past?'^28^ which can also reduce feelings of discomfort and stress.

### During the treatment

There was one paper providing a communication phrase to use during the treatment.^22^ OH practitioners are to use consent-based communication, provide clear and honest explanations of procedures, their rationale, expected sensations, and estimated duration to practice TIC.^22^^,^^31^^,^^33^ Explaining the procedure and reaffirming consent throughout the treatment can create sense of safety and trust when practising TIC.

### When trauma signs and symptoms are recognised

OH setting and treatments can be challenging for patients with trauma. Common situations such as lying down under an authority figure, the smell of latex gloves, having a bib around the neck or water in the mouth can trigger memories of past traumatic events and re-traumatise patients with behavioural dysregulation and increased risk of dental fear and anxiety.^28^^,^^29^^,^^34^ Lapidos *et al.*^35^ recommend reflecting this understanding and validating a patient's experience by making a statement such as ‘it is common to have a stress reaction or to experience anxiety in our setting'^34^ and ask ‘how can we make this experience better for you?'.^35^

Patients with trauma may also dissociate during dental treatment, where they feel detached from their body and the present moment. Patients experiencing dissociation may present with sudden sensory loss, emotional withdrawal, unresponsiveness, anger, seizure-like convulsions, or transient paralysis, with observable shifts in facial expression and demeanour.^29^ Two papers make suggestions when communicating with patients who have dissociated.^29^^,^^31^ Dissociative reactions are confusing and can be frightening to professionals outside of behavioural science. However, OH practitioners cannot ignore the signals and continue treatment while the patient is not mentally present. When unsure, Anstorp *et al.*^29^ suggested asking directly ‘some patients are disconnecting or dissociate during dental treatment, is this something you think may happen to you?'. If the patient responds that this could become a problem, then following was recommended, ‘is there anything special we should look for as a sign that you are about to unwind?'.^29^

### When trauma history is disclosed

While trauma disclosure is not necessary to practice TIC, literature acknowledges that patients can disclose their trauma history in OH settings and three papers provided communication phrases to practise TIC when trauma history is disclosed.^22^^,^^28^^,^^35^ When the patient shares their trauma experience, OH practitioners should thank the patient and focus on how the team can support the patient to feel more comfortable.^28^

Validating statements and questions^22^^,^^35^ can help create sense of safety and trust, empower the patients and help patients and OH practitioners understand the current struggles with greater empathy and guide patients to resources and support.

### Active listening

Active listening in healthcare is a critical communication skill that enhances the empathetic relationship between practitioners and patients, leading to improved clinical outcomes, patient satisfaction, and adherence to treatment plans.^36^^,^^37^ Active listening is essential when providing TIC, as it promotes psychological safety, trust, and emotional validation, core principles of TIC practice. By demonstrating empathy and attunement, OH practitioners can help mitigate perceived threats and loss of control, enabling patients to communicate their concerns more openly.^22^^,^^28^^,^^29^^,^^32^^,^^38^ While a considerable number of papers discussed the importance of active listening in TIC, only one paper presented a phrase for active listening with reflecting questions and statements.^31^ Through active listening, demonstrating genuine attention, empathy, and validation, OH practitioners can communicate respect and understanding, which helps to reduce perceived threat and re-establish a sense of control supporting TIC practice, reducing the risk of re-traumatisation.

### Motivational interviewing

Motivational interviewing (MI) in healthcare is a patient-centred communication approach, which has been found to be an effective intervention for change.^39^ In OH, MI has been associated with enhanced practitioner confidence, professionalism, and therapeutic rapport, alongside improved patient outcomes across diverse OH conditions and preventive care.^40^ One paper offered communication phrases when using MI for TIC practice. A MI technique called important rulers can be used to gauge patients' level of desire and confidence for change. Question such as ‘on a scale of 1–10, how important is it to you to improve the health of your gums?'^30^ can encourage patients to express their own motivations and readiness for change, rather than feeling pressured or judged, which helps to reduce perceived power imbalances for patients with trauma. MI can mitigate anxiety, enhance engagement with OH care, and support gradual behaviour change without re-traumatisation.

## Discussion

This review identified communication phrases which can be used by OH practitioners when practising TIC categorised by specific clinical context: before the treatment, screening for trauma, during the treatment, when trauma signs and symptoms are recognised, when trauma history is disclosed, active listening and motivational interviewing. Our findings demonstrate that TIC is not a distinct or isolated ‘event' but rather a continuous process embedded throughout patient care through the use of consent-based communication to support the five pillars of TIC, safety, trust, control, collaboration and empowerment. With limited established TIC practices in OH, identified TIC communication phrases could support OH practitioners towards creating a psychologically safe environment for patients with trauma to reduce the risk of re-traumatisation and unnecessary distress for both patients and OH practitioners.

There are several studies which have researched communication phrases in health care for both common and unfamiliar or challenging clinical situations. In nursing, script-based communication has been advocated to enhance patient empowerment, engagement, and activation.^15^ Hashim^41^ proposed communication phrases for patient-centred medical interviewing to help elicit the patient's perspective, to express empathy to patients, for revealing a new diagnosis or breaking bad news for family physicians. Clancy *et al.*^9^ further noted that emotionally charged or serious clinical situations can increase the level of stress which can impair practitioner's ability to respond rationally or articulate appropriate language. and structured communication phrases may assist in managing challenging, uncertain, or distressing encounters. OH settings are often private and intimate environments, where patients with a history of trauma may be inadvertently triggered during care, and managing such patients can be emotionally demanding for OH practitioners. TIC communication phrases identified in this review may therefore support the practical implementation of TIC within OH practice.

Scripted communication may offer structure and consistency. However, its overreliance may fail to accommodate the individual and situation-sensitive nature of healthcare interactions. According to Honeycutt and Sheldon,^42^ scripts are expectation-based cognitive frameworks guiding communication that may become mismatched between individuals leading to misinterpretation and dysfunctional interactions. Inappropriately applied communication scripts may render communication forced, mechanical or insincere.^9^ Accordingly, communication should remain flexible, sincere, respectful and empathetic to ensure that communication phrases are conveyed with authenticity. When implemented with this awareness, TIC communication phrases may serve as a valuable resource for OH practitioners to practice unfamiliar TIC in everyday clinical encounters. Furthermore, while this review focused on communication phrases alone, it is important to recognise that TIC encompasses a broader framework beyond conversational skills. The communication phrases identified in this review should be regarded as a foundational basis for development of comprehensive TIC education and training in OH.

Most of the identified TIC communication phrases can be readily integrated into routine clinical practice to foster a psychologically safe environment for all patients, ensuring that patients are not required to disclose sensitive personal information in order to receive empathetic, safe, trusting, and empowering care. However, phrases intended for trauma screening may be more challenging for OH practitioners to implement with concerns including potential intrusiveness, time constraints and insufficient resources and training to manage the significant emotional responses that screening may elicit.^22^^,^^25^^,^^32^ Nevertheless, screening for trauma is generally recommended for populations with high risk of trauma or/and with evidence of physical injuries. To minimise potential intrusiveness, the practitioner should first prioritise rapport-building and development of a trusting relationship with the patient, provide clear rationale for screening as a part of duty of care and explicitly address the relationship between overall wellbeing and OH care. When trauma is disclosed, OH practitioners must be cognisant of their legal obligations regarding mandatory reporting in cases of suspected abuse, in accordance with the jurisdiction in which they practice.

While practitioner-patient communication is fundamental to quality patient care, effective TIC implementation requires its adoption at all levels of an organisation.^16^ Communication phrases identified in the review are primarily directed towards OH practitioners. However, communication by other staff members can also significantly influence the care experience of patients with trauma. Jackson and McLernon^34^ recommends administration staff to support patients with trauma by asking questions such as ‘do you find the dental environment difficult?' or ‘is there anything the dentist should be aware of before the appointment?' when booking appointments to better understand the needs and potential triggers from patients before their first appointment. The development of TIC communication phrases for the entire dental team may therefore enhance the overall standard of care and support more effective TIC implementation in OH.

### Limitations and future research

This review has identified published TIC communication phrases; however, there are no studies conducted evaluating the effectiveness of communication phrases in the implementation of TIC. Future studies examining OH practitioners' perceived ease of use, TIC competence, and attitudes, alongside patients' perceptions of care when such communication strategies are employed, would provide a valuable and practical avenue for assessing their effectiveness and improve TIC implementation in OH.

## Conclusion

This review identified communication phrases for OH practitioners when practising TIC. The findings provide practical, readily applicable tools aligned with core TIC principles; safety, trust, control, collaboration, and empowerment, aimed at minimising the risk of re-traumatisation in OH settings. While communication must to be tailored to individual patient needs, familiarity with TIC communication phrases could support OH practitioners to enhance the overall patient experience and reduce the burden and challenges associated with practising unfamiliar TIC.

## Data Availability

Available within the paper.
